# Deletion of Ultraconserved Elements Yields Viable Mice

**DOI:** 10.1371/journal.pbio.0050234

**Published:** 2007-09-04

**Authors:** Nadav Ahituv, Yiwen Zhu, Axel Visel, Amy Holt, Veena Afzal, Len A Pennacchio, Edward M Rubin

**Affiliations:** 1 Genomics Division, Lawrence Berkeley National Laboratory, Berkeley, California, United States of America; 2 United States Department of Energy Joint Genome Institute, Walnut Creek, California, United States of America; Baylor College of Medicine, United States of America

## Abstract

Ultraconserved elements have been suggested to retain extended perfect sequence identity between the human, mouse, and rat genomes due to essential functional properties. To investigate the necessities of these elements in vivo, we removed four noncoding ultraconserved elements (ranging in length from 222 to 731 base pairs) from the mouse genome. To maximize the likelihood of observing a phenotype, we chose to delete elements that function as enhancers in a mouse transgenic assay and that are near genes that exhibit marked phenotypes both when completely inactivated in the mouse and when their expression is altered due to other genomic modifications. Remarkably, all four resulting lines of mice lacking these ultraconserved elements were viable and fertile, and failed to reveal any critical abnormalities when assayed for a variety of phenotypes including growth, longevity, pathology, and metabolism. In addition, more targeted screens, informed by the abnormalities observed in mice in which genes in proximity to the investigated elements had been altered, also failed to reveal notable abnormalities. These results, while not inclusive of all the possible phenotypic impact of the deleted sequences, indicate that extreme sequence constraint does not necessarily reflect crucial functions required for viability.

## Author Summary

It is widely believed that the most evolutionarily conserved DNA sequences in the human genome have been preserved because of their functional importance and that their removal would thus have a devastating effect on the organism. To ascertain this we removed from the mouse genome four ultraconserved elements—sequences of 200 base pairs or longer that are 100% identical among human, mouse, and rat. To our surprise, we found that the mice lacking these elements are viable, fertile, and show no apparent abnormalities. This completely unexpected finding indicates that extreme levels of DNA sequence conservation are not necessarily indicative of an indispensable functional nature.

## Abstract 3

Ultraconserved genomic elements may be conserved because they are functional. Here, a number of these elements have been deleted in the mouse, and animals are viable, suggesting that these sequences are dispensable.

## Introduction

Evolutionary conservation has become a powerful means for identifying functionally important genomic sequences [1,2]. Ultraconserved elements have been defined as a group of extremely conserved sequences that show 100% identity over 200 bp or greater between the human, mouse, and rat genomes [3]. This category of extreme evolutionary sequence conservation is represented by 481 sequences in the human genome, of which over half show no evidence of transcription. Further analysis of the distribution of these noncoding ultraconserved elements demonstrates that they tend to cluster in regions that are enriched for transcription factors and developmental genes [3], and a limited number of functional studies suggest a role for some of these noncoding elements in gene regulation [4–6].

Several hypotheses have been proposed to explain the extreme sequence constraint of ultraconserved elements, including strong negative selective pressure and/or reduced mutation rates [3]. The negative selection hypothesis postulates that crucial functions such as vital gene regulatory information is embedded within these sequences, while the reduced mutation rate hypothesis suggests that these sequences exist in a hyperrepaired or hypomutable state [3]. Recent analysis of human variation in these noncoding ultraconserved elements provides compelling evidence supporting negative selection as contributing to their extreme evolutionary conservation [7]. Furthermore, noncoding ultraconserved elements have also been shown to be significantly depleted in human segmental duplications and copy number variants, suggesting that disruption of their normal copy number may lead to reduced fitness [8]. In this study, we removed four carefully chosen noncoding ultraconserved elements in the mouse genome to directly explore a functional role for these elements in vivo.

## Results

### Generation and General Characterization of Ultraconserved Knockout Mice

**Figure 1:**
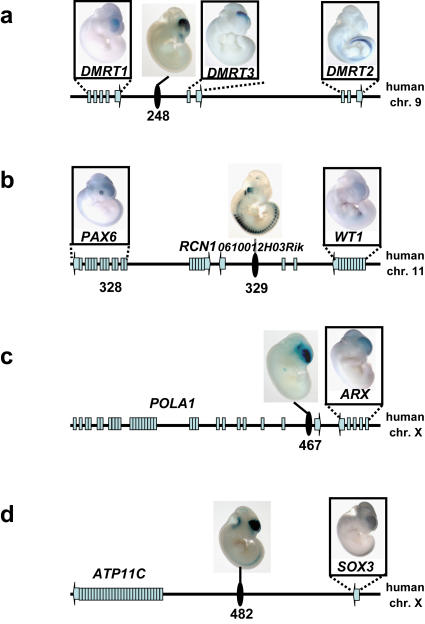
Schematic of the Human Genomic Locations of the Four Ultraconserved Elements That Were Deleted (A) uc248 region; (B) uc329 region; (C) uc467 region; (D) uc482 region. A black oval represents each ultraconserved element, while the embryos above the schematics represent observed positive enhancer activities captured through transgenic mouse testing at e11.5 for that element [6]. Stained embryos in boxes represent whole-mount in situ hybridizations of the specific gene at e11.5 (genes without stained embryos were negative for this assay at this time point). Exons and noncoding elements not shown to scale.

To increase the probability of observing an associated phenotype in the ultraconserved null mice, we employed a variety of criteria in selecting the noncoding ultraconserved elements for deletion. We chose elements that showed tissue-specific in vivo enhancer activity in a mouse transgenic reporter assay that tended to recapitulate aspects of the expression pattern found in genes that were in their proximity (Figure 1) [6]. Other factors that were taken into account in prioritizing elements for deletion included their proximity to genes whose inactivation or alteration in expression result in specific phenotypes that we could screen for in the ultraconserved element deletion mice (Table 1). Elements meeting most of these criteria were chosen for removal and included: uc248, uc329, uc467, and uc482 (Figure 1) [3], representing 222, 307, 731, and 295 bp, respectively, of 100% identity between human, mouse, and rat.

**Table 1:**
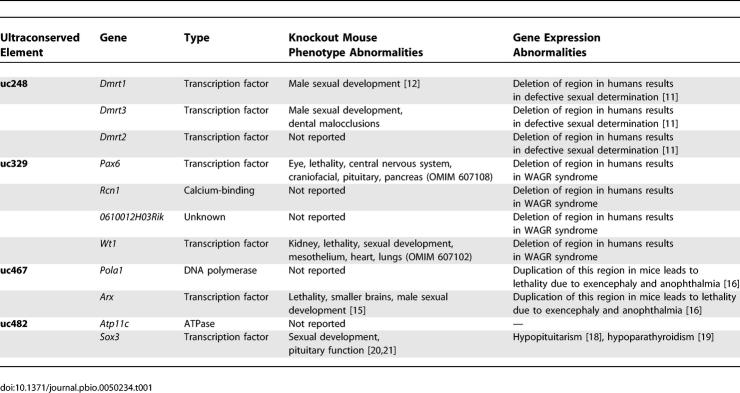
Mouse Knockout and Gene Expression Phenotypes for Genes Adjacent to the Deleted Ultraconserved Elements

**Figure 2:**
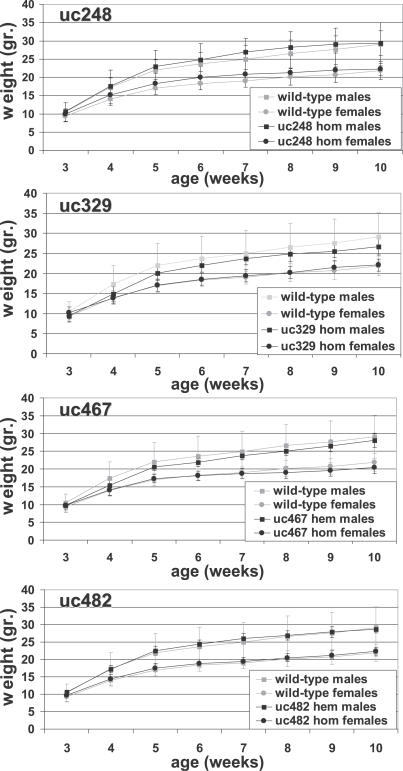
Growth Curves of Each Ultraconserved Element Knockout Mouse Strain Error bars indicate standard deviation. hem, hemizygous; hom, homozygous.

All four noncoding ultraconserved elements were deleted from the mouse genome using standard mouse genetic engineering techniques, and removal was confirmed by PCR and Southern blot hybridization (Protocol S1). We first examined each line for the viability of homozygous/hemizygous knockout mice in mixed crosses, and observed that all four lines showed no reduction in the expected number of homozygous/hemizygous mice that were generated (Tables 2 and 3). Homozygous matings within the four lines revealed no significant differences in viability and litter size compared to the wild-type littermates (Table 4). We next examined body weight (up to 10 wk of age; Figure 2) and survival (up to 25 wk; see Materials and Methods), and found no significant differences compared to the wild-type littermates. Further analysis of a standard panel of 16 different clinical chemistry assays in each of the mouse lines detected only a few small differences compared to the wild-type littermates (Figure S1). Expression analysis of genes adjacent to each element by whole-mount in situ hybridization at embryonic day 11.5 (e11.5) revealed no apparent differences between null embryos and their wild-type littermates, except for a moderate reduction in forebrain expression of *SRY-box containing gene 3 (Sox3)* in uc482 null embryos (Figure S2). Quantification by real-time PCR suggested a slight reduction in *Sox3* e11.5 head expression that was, however, insignificant (29.63 in wild-types compared to 23.66 in nulls, corresponding to 18S RNA expression; *p* = 0.64, unpaired *t*-test). General pathological analysis of 6-wk-old mice revealed no distinct differences compared to the wild-type littermates (Table S1), with one exception. The exception was one uc329 homozygous male having unilateral renal agenesis. Additional analysis of 102 uc329 homozygous null mice revealed a total of two mice (including the initial propositus) with one kidney, compared to none within the 30 uc329 wild-type littermates that were screened. Unilateral renal agenesis is estimated to occur in 1 to 1,000 live births in humans [9] and is asymptomatic and unassociated with a reduction in survival rate [10]. Possible explanations for unilateral renal agenesis in ∼2% of uc329 homozygous null mice in this study include a spontaneous event unassociated with the deleted element or a low penetrance phenotype caused by the absence of this element.

**Table 2:**
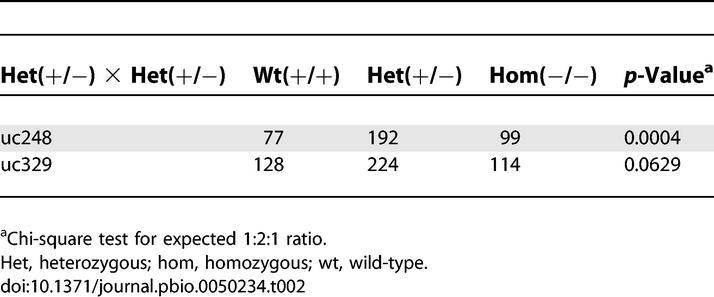
Summary of Observed Offspring from Heterozygous × Heterozygous Mouse Crosses

**Table 3:**
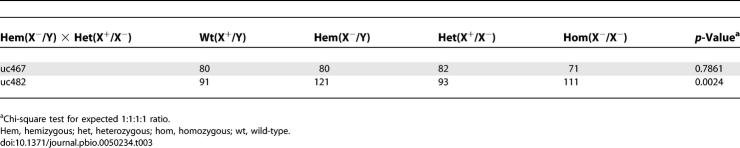
Summary of Observed Offspring from Hemizygous × Heterozygous Mouse Crosses

**Table 4:**
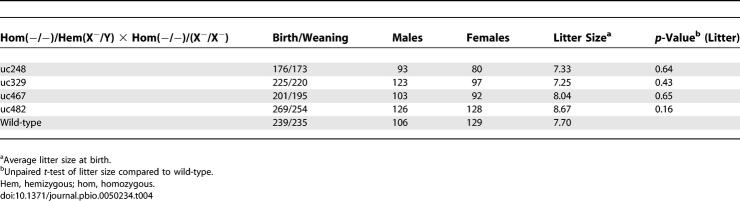
Summary of Observed Offspring from Homozygous/Hemizygous × Homozygous Mouse Crosses

### Screens for Phenotypes of Adjacent Genes

In addition to the above general screens, we screened each of these mouse lines for phenotypes specifically associated with the inactivation or dosage abnormality of the genes in proximity to the deleted ultraconserved elements. uc248 is bracketed by the genes *doublesex and mab-3 related transcription factor 1 (DMRT1)* and *doublesex and mab-3 related transcription factor 3 (DMRT3)* (Figure 1A). In humans, haploinsufficiency due to chromosomal aberrations within this region leads to XY sex reversal [11]. In mice, *Dmrt1* homozygous knockouts exhibit defects in testicular development [12], while *Dmrt3* function is unknown. In order to identify the phenotype associated with *Dmrt3* deficiency for these studies, we deleted *Dmrt3* from the mouse genome. All *Dmrt3* null homozygous mice died from starvation at 2 mo of age due to dental malocclusions, and in addition some of the males exhibited male sexual development abnormalities (N. Ahituv, unpublished data). Based on these results, we extensively phenotyped uc248 homozygous null mice for sexual and dental abnormalities. Pathological analysis of both male and female sexual organs and teeth in 6-wk-old uc248 null mice revealed no obvious defects (Table S1). In addition, heterozygous and homozygous crosses exhibited no reduction in expected homozygous offspring (Tables 2 and 4).

uc467, the longest solitary noncoding ultraconserved element in the human genome (731 bp), lies inside the last intron of *polymerase (DNA directed), alpha 1 (POLA1)* adjacent to the *aristaless related homeobox (ARX)* gene (Figure 1C). Mutations in *ARX* in humans lead to a wide range of neurological and sexual development disorders [13,14], while hemizygous *Arx* null male mice die shortly after birth and have small brains and male sexual development abnormalities [15]. In addition, a duplication of this region in mice, caused by insertional mutagenesis, leads to embryonic lethality due to exencephaly accompanied by anophthalmia [16]. Detailed pathological examination of the reproductive organs and neuroanatomical examination of the brains of uc467 null mice revealed no apparent abnormalities (Table S1). In addition, the mice showed no obvious differences in the offspring expected from the hemizygous × heterozygous and hemizygous × homozygous crosses (Tables 3 and 4).

uc329 lies in the middle of an 80-kb intronic region of the hypothetical gene *0610012H03Rik* in a region adjacent to the *reticulocalbin 1, EF-hand calcium binding domain (RCN1)* gene and two developmental transcription factors, *Wilms tumor 1 (WT1)* and *paired box 6 (PAX6)* (Figure 1B). Mutations in humans in *WT1* and *PAX6* respectively cause Wilms tumor and type 2 aniridia, while chromosomal deletions encompassing all four genes lead to WAGR syndrome. Mouse knockouts generated for *Wt1* and *Pax6* have a variety of phenotypes, the most notable being kidney and eye abnormalities, respectively. Detailed pathological analysis of the kidneys and eyes of the uc329 null mice revealed no significant differences compared to the wild-type littermates (Table S1), other than the ∼2% unilateral renal agenesis discussed above. Clinical chemistry tests revealed slightly higher urea nitrogen levels compared to the wild-type littermates (33.16 versus 26.16 mg/dl; *p* = 0.032, unpaired *t*-test; Figure S1), while creatinine levels, which are a more specific measure for kidney function, were similar to those in the wild-type littermates (0.25 versus 0.22 mg/dl; *p* = 0.165, unpaired *t*-test; Figure S1).

uc482 resides in a gene desert between the *ATPase, Class VI type 11C (ATP11C)* and *SRY (sex determining region Y)-box 3 (SOX3)* genes (Figure 1D). Human *SOX3* mutations lead to X-linked mental retardation with isolated growth hormone deficiency [17] and hypopituitarism [18], while *SOX3* dosage defects are suggested to cause hypopituitarism [18] and hypoparathyroidism [19]. In mice, deletion of *Sox3* results in sexual development and pituitary abnormalities [20,21]. Pathological analysis of the reproductive organs of uc482 null mice revealed no significant abnormalities (Table S1), and hemizygous × heterozygous and hemizygous × homozygous crosses exhibited no reduction in expected homozygous/hemizygous offspring (Tables 3 and 4). Growth hormone abnormalities would be expected to lead to body weight irregularities, none of which were detected (Figure 2). Calcium levels were also normal (Figure S1), supporting a lack of marked abnormalities in parathyroid gland function.

## Discussion

Based on the compelling evidence that ultraconserved elements are conserved due to functional constraint, it has been proposed that their removal in vivo would lead to a significant phenotypic impact [7,8]. Accordingly, our results were unexpected. It is possible that our assays were not able to detect dramatic phenotypes that under a different setting, for instance, outside the controlled laboratory setting, would become evident. Moreover, possible phenotypes might become evident only on a longer timescale, such as longer generation time. It is also possible that subtler genetic manipulations of the ultraconserved elements might lead to an evident phenotype due to a gain-of-function-type mechanism. All four elements examined in this study demonstrated in vivo enhancer activity when tested in a transgenic mouse assay (Figure 1) [6], which would suggest regulatory element redundancy as another possible explanation for the lack of a significant impact following the removal of these specific elements. Just as gene redundancy has been shown to be responsible for the lack of phenotypes associated with many seemingly vital gene knockouts, regulatory sequence redundancy [22] can similarly provide a possible explanation for the lack of a marked phenotype in this study. While our studies have not defined a specific need for the extreme sequence constraints of noncoding ultraconserved elements, they have ruled out the hypothesis that these constraints reflect crucial functions required for viability.

## Materials and Methods

### Generation of ultraconserved element null mice.

The basic technology used for gene targeting and screening has been described previously [23]. Briefly, the four selected ultraconserved elements were removed in W4/129S6 mouse embryonic stem cells (Taconic, http://www.taconic.com/) by standard replacement of a LoxP-flanked neomycin cassette. To avoid potential regulatory effects due to the neomycin gene cassette, we subsequently removed it by Cre-mediated recombination of LoxP sites in the embryonic stem cells. All positive colonies in each stage were confirmed by PCR and Southern analysis (Protocol S1) and then injected into C57BL/6J blastocyst stage embryos. Chimeric mice were subsequently crossed to C57BL/6J mice, generating agouti offspring that were heterozygous/hemizygous for the ultraconserved element deletion and were intercrossed to generate homozygous ultraconserved null mice. Genotyping was carried out using standard PCR techniques (Protocol S1).

### Survival.

Eight males and eight females from each line and wild-type littermates were analyzed for survival up to 25 wk. Mice were housed in a temperature-controlled room under a 12-h light/dark cycle, given free access to water, and fed ad libitum on a standard chow. No lethality was observed for any of the strains during the period of study.

### Clinical chemistry.

Serum samples from at least six males and six females at 10–14 wk of age from each line were analyzed using the automated spectrophotometric chemistry analyzer Hitachi 917 at Marshfield Laboratories (http://www.marshfieldlaboratories.org/) following standard protocols.

### Whole-mount in situ hybridization.

Four e11.5 wild-type embryos were analyzed for each gene. Genes that were positive for expression at this time point were further analyzed for expression differences using four homozygous null and four wild-type littermates at e11.5. Briefly, embryos were fixed overnight in 4% paraformaldehyde followed by methanol washes. Whole-mount RNA in situ hybridization was carried out using standard protocols [24] with antisense digoxigenin-labeled riboprobes. The following vectors were used as templates for probes: *Dmrt1* (kind gift from D. Zarkower, University of Minnesota), *Dmrt2* (IMAGE 1248080, http://image.llnl.gov/), *Dmrt3* (IMAGE 6404988), *Pax6* (IMAGE 4504106), *Rcn1* (IMAGE 6414128), *0610012H03Rik* (IMAGE 5042053), *Wt1* (RNA probe 777, GenePaint.org, http://genepaint.org/), *Pola1* (IMAGE 894396, 30063811, and 30103897), *Arx* (IMAGE 5707995), *Atp11c* (IMAGE 30843359), and *Sox3* (IMAGE 5717161). Stained embryos were analyzed using a Leica (http://www.leica.com/) MZ16 microscope and photographed with a Leica DC480 camera.

### Real-time quantitative PCR.

Total RNA was extracted using TRIzol reagent (Invitrogen, http://www.invitrogen.com/) from the heads of four uc482 homozygous/hemizygous null and four wild-type littermates at e11.5, and pooled separately. Following reverse transcription with SuperScript First-Strand Synthesis System (Invitrogen), real-time PCR was performed using *Sox3*-specific primers (forward: agcgcctggacacgtacac; reverse: atgtcgtagcggtgcatct), QuantumRNA Universal 18S (Ambion, http://www.ambion.com/), and the SYBR Green PCR Master Mix (Applied Biosystems, http://www.appliedbiosystems.com/) on a 7500 Fast Real-Time PCR System (Applied Biosystems). All procedures and calculations were carried out according to manufacturers' recommendations.

### Necropsy and pathology examination.

Two male and two female 6-wk-old mice from each knockout line and wild-type littermates were submitted to the Comparative Pathology Laboratory at the University of California Davis. Tissues were fixed in 10% phosphate buffered formalin for at least 24 h and processed using routine methods to Hematoxylin-and-Eosin-stained sections and subsequently analyzed for any abnormalities.

## Supporting Information

Figure S1 Clinical Chemistry Analysis in Ultraconserved Knockout Mice Bar charts represent the average parameters for each line; error bars indicate standard deviation. An asterisk indicates *p* <0.05 in an unpaired *t*-test compared to the wild-type littermates.
(968 KB EPS)

Figure S2 Whole-Mount In Situ Hybridization Analysis of Homozygous/Hemizygous Knockouts and Wild-Type Littermates Representative embryos for each gene that is positive for this assay at e11.5 are shown. The red arrows indicate the *Sox3* forebrain expression in uc482 embryos.
(7.7 MB TIF)

Protocol S1 Supplementary Methods (275 KB PDF)

Table S1 Summary of Pathological Analysis in Ultraconserved Knockout Mice (83 KB PDF)

### Accession Numbers

The Entrez Gene (http://www.ncbi.nlm.nih.gov/sites/entrez?db=gene) accession numbers for the mouse genes discussed in this paper are *Arx* (11878), *Atp11c* (54668), *Dmrt1* (50796), *Dmrt2* (226049), *Dmrt3* (240590), *Pax6* (18508), *Pola1* (18968), *Rcn1* (19672), *Sox3* (20675), and *Wt1* (22431). The Entrez Gene accession numbers for the human genes discussed in this paper are *ARX* (170302), *ATP11C* (286410), *DMRT1* (1761), *DMRT2* (10655), *DMRT3* (58524), *PAX6* (5080), *POLA1* (5422), *RCN1* (5954), *SOX3* (6658), and *WT1* (7490). The GenBank (http://www.ncbi.nlm.nih.gov/Genbank/) accession number for hypothetical gene *0610012H03Rik* is NM_028747. The OMIM (http://www.ncbi.nlm.nih.gov/sites/entrez?db=OMIM) accession numbers for the genetic disorders discussed in this paper are type 2 aniridia (106210), WAGR syndrome (194072), and Wilms Tumor (194070).

## Abbreviations

e11.5: embryonic day 11.5
