# Supplementary material for: Deletion of Ultraconserved Elements Yields Viable Mice
Source: PLoS Biol. 2007 Sep 4;5(9):e234. doi: 10.1371/journal.pbio.0050234 (PMC1964772; doi:10.1371/journal.pbio.0050234)
Supplement: Supplementary file 1 [file pbio.0050234.sd001.pdf]

Supplementary Methods

Generation of ultraconserved element null mice

Homologous arms were amplified by PCR (see primer tables below for each element) from DNA extracted from W4/129S6 mouse embryonic stem cells (Taconic) and cloned into the ploxPN2T vector. ploxPN2T was modified from pPN2T [1] by replacing the existing neomycin selection cassette with a neomycin selection cassette flanked by *loxP* sites for positive selection and two hsv-tk cassettes for negative selection. Constructs were linearized and 20 μg were electroporated into W4/129S6 mouse embryonic stem cells. The electroporated cells were selected under G418 (160 μg/ml) and 0.2 μM FIAU for a week. Colonies that survived were picked and expanded on 96-well plate and screened both by PCR with primers outside but flanking the homologous arm, and by Southern blot hybridization with probes targeting the vicinity of the homologous arms. To avoid potential regulatory effects due to the neomycin gene cassette, we next removed it by Cre-mediated recombination of LoxP sites in the ES cells. Clones that were correctly targeted were electroporated with 20 μg of the Cre recombinase-expressing plasmid TURBO-Cre (Obtained from Dr. Timothy J. Ley of the Embryonic Stem Cell Core at the Siteman Cancer Center, Washington University Medical School). Clones positive for Neo removal were screened both by PCR (primers and gels for each element below) and Southern blot hybridization (gel results below for each element), and checked for G418 sensitivity. Positive colonies were injected into C57BL/6J blastocyst stage embryos. Chimeric mice were subsequently crossed to C57BL/6J mice, generating agouti offspring that were heterozygous/hemizygous for the ultraconserved element deletion and were intercrossed to generate homozygous ultraconserved null mice.

Genotyping

Genomic DNA was extracted from a 1-cm section of tail that was incubated overnight in lysis buffer (containing 50mM Tris-HCl pH 8.0, 1mM EDTA, 1% SDS, 20mM NaCl and 1mg/ml Proteinase K) at 55 degrees Celsius. Genotyping was performed by PCR, with primers outside the deleted segments and within the targeting vector (see primer tables below for each element).

Figure legend for genomic targeting and screening below.

Design of ultraconserved deletions displaying the wild-type, the Neo insertion (+Neo) and the Neo deletion (DEL) allele for each element (figures not drawn to scale). The points of integration of target vector homology arms are shown, primers (P2/P3.fwd and P2/P3.rev) used to screen embryonic stem (ES) cells by PCR for the presence of the Neo cassette and the Neo cassette deleted cells are indicated by arrows flanking the deleted segment. The probe used in the Southern hybridization for identification of embryonic stem cells carrying the deletion is shown as a striped box, together with the restriction sites. The sequence that was deleted, the expected DNA sizes for the Southern hybridization and PCR, the Southern hybridization and PCR gel results of each allele, and the primers that were used are shown following the figure.

uc248 deletion

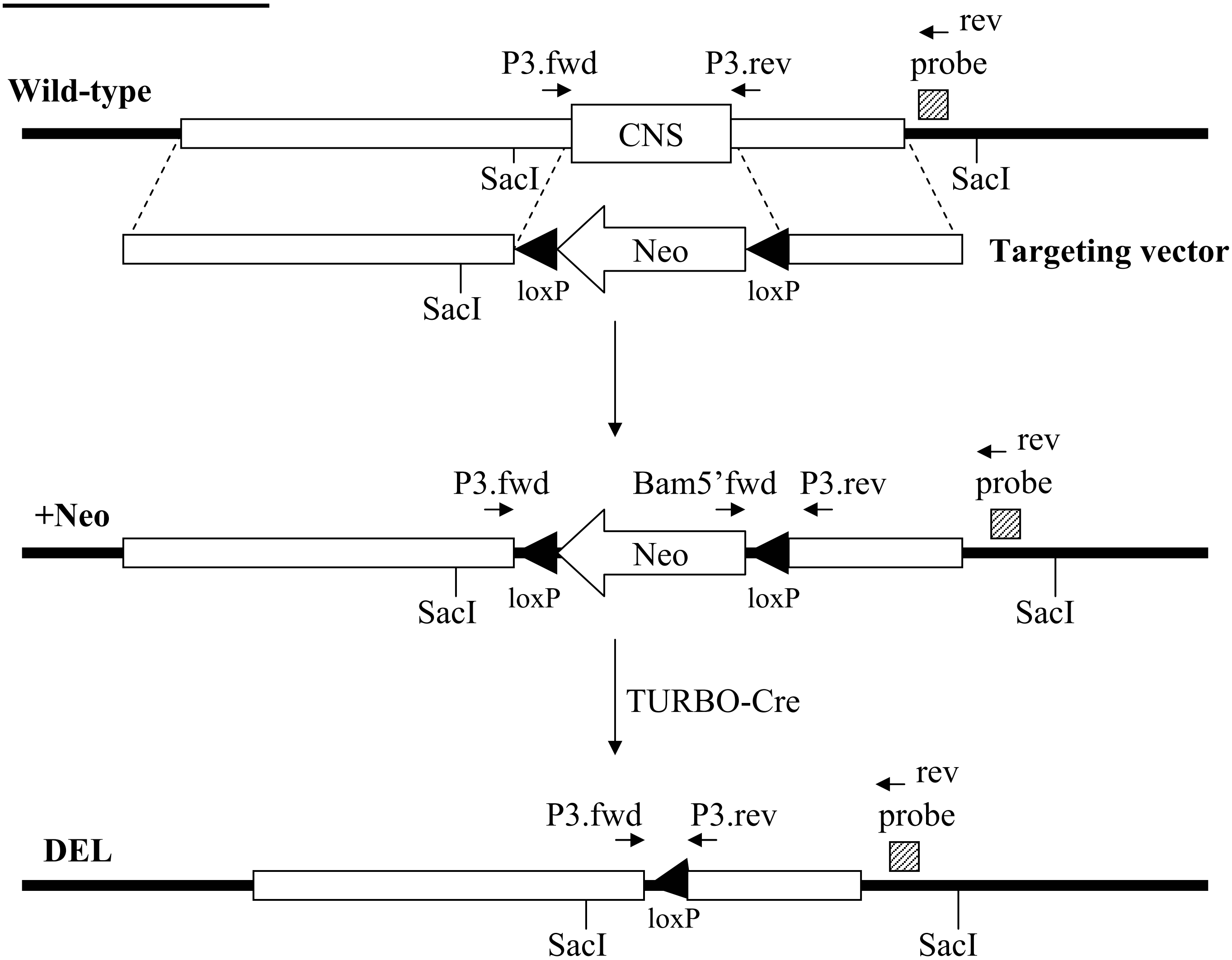

-Figure legend on page 2.

uc248 deleted sequence

gagtattaatacctgccatatcttaccgggcgaattgtggtttgaaaacactagcacaccactcagaggagctgggagtgggtgtcgtctgtgaggttacttt
cacttgtaaccctccccctccctgtccaatttctttgatgatttttcccataaatgcataagctgcagggacttagtagaagcaggccgcgccccctaaatgtg
attcggaccatatgctttctacattcatccccgcatgggcttttcttctctgaactaactgctaacacgttatagtcaccttgaaatttcctctgctattttccaatta
aaagcttaatagccctggaaacggagttcatgtggtgaagtttaccatagccccttgttctgaaaggctttctgctgggatcccattatgtgcttgaataaacc
ctttctgcaaacagaaatgggactcggggttgtcaggtgttgggttacaatagactttgaggcaggggacattttaccaacataaatacaaacctgtccctat
agggagatgttgtcaaatactgggatatggaaaacattcccatcaaatatgagaaaaggattaccgcgctatatcaaatgagtatttagatctcggccctcg
atgtgcactggagtgaaacggggcccagtaccaaataataccagagtgtgtgagtggcactgggatagggtggctaggtgacccaggaaggaaaggg
gattgctttgagaatgaaaatgtcccctaaaaacc

Southern or PCR	Wild-type	Neo targeted	Neo deleted	Screen for
SacI digestion	3247 bp	4525 bp	2573 bp	Both
PCR(Bam5'.fwd/rev)	No band	1749 bp	No band	Neo targeted
PCR (P3.fwd+P3. rev)	905 bp	2183 bp	231 bp	Neo removed

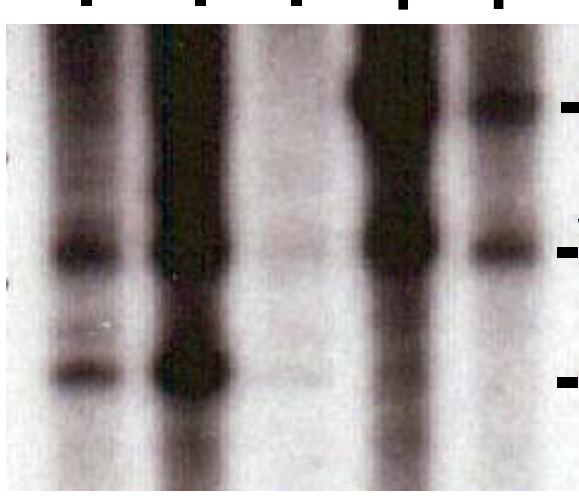

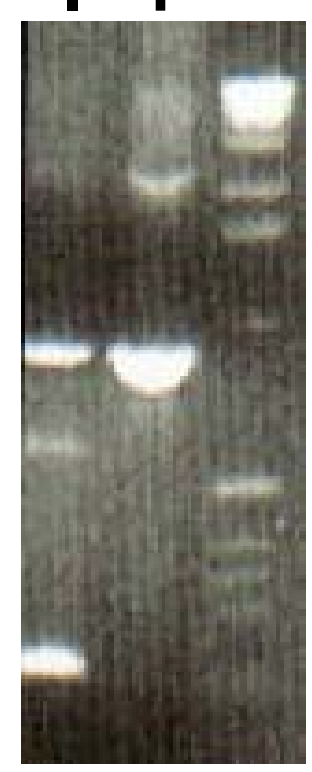

uc248 Primers

	Primers	Size (bp)
Long arm	5':gaatgcggccgccagaactgagttgtcaggaaactgtct	6039
	3':acgcgtcgacaagtggctgaaacaacgtcgtgctt	
Short arm	5': ccatcggatcctggtgcagaactctgctgctctct	1602
	3': atcggcaattgagcttgcgagggaattgagggtac	
Probe	5': aagggtgggaaggaaagcggtcta	311
	3': tttcctcctccctggcaggca	
Neo target screening	5': ttggctggacgtaaactcctctt	Neo-1749<br>WT-none
	3': tagaccgctttccttcccaccctt	
Neo removal	P3.fwd.: agcttccgatgttcacgttgt	Neo del-231<br>Neo-2183<br>WT-905
	P3.rev: tggtcagggaaacagatcagg	
Genotyping	P3.fwd: agcttccgatgttcacgttgt	Del-231<br>WT-321
	P3.rev: tggtcagggaaacagatcagg	
	3': tcctctgagtggtgtgctagtgtt	

uc329 deletion

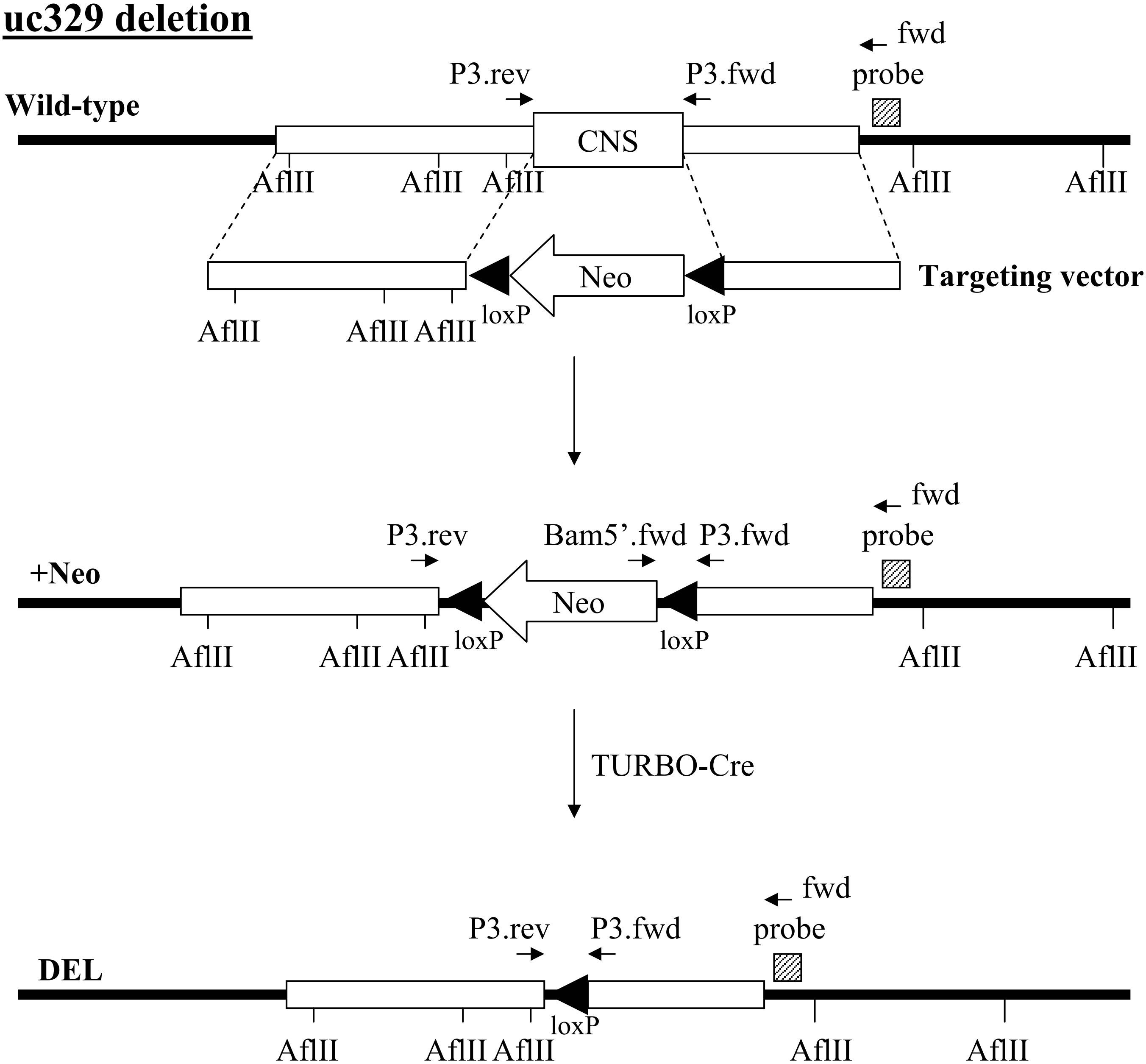

-Figure legend on page 2.

uc329 deleted sequence

ccaccctggccagcttgctcagaaagcagcgttctctccgagttcctctgcctctgttctctggcccttttggcttgagatgttttctatcgtttctatatttcaaa
gctttgtggtaaataatcaattcaaaccagcaccgagtctctctttctgtaattctaaaaacccattaaattatattgttgggaggaaacaatagctgtgctaatc
acgctctgtgcatgcgaatcttaatttccccagaggcctgaatatgaatttttactaacatgttaattttgtgtgttaatcagtagctgtaggccgaatgaatgga
ttgactatgtaaaactgtttgatattgaaacaactccttttgaccccctcaaattggcttcattcccctaaagacggttaaagttaattaagcactcaataggttta
tggtgtttaaattaaaatatattactcctcattgtgtagtcatcaagtgagaaagacatcctcctcctggctgcctggctggctaatgggcttatcaaaattcatg
atgtaataagaaggccgacaagtgcatatgagagtctggctgtcactcaaggaaaaacactgaccatcttttcccactagagggcagaagaaagccaca
aatggccacactggcgctgtagtctgaggaattggaggaggatagcccctcaaaggcatttgtaaacaaaatattttgttaccatccccgcccccacccag
tgcatagccctgtcccagtttgcccagttcctcctcttccctgtcacatggtctacctcctcggatgcccttgtgactgtaaattaacaggaaagggttggaga
gatggcttcagggttaagagcacttgctgttcttgcagtgggcccaggttcagttcccagtacccacaccaggtcaccaagtgactcacagttccaggctg
tttgatgctatcttctggcctccaagagcacctgttaaacacacacacacacacacacacacacacacacacacacacacgtttttggttttgtttttaact
at

Southern or PCR	Wild-type	Neo targeted	Neo deleted	Screen for
AflII digestion	4106 bp	5097 bp	3145 bp	Both
PCR(Bam5'.fwd/fwd)	No band	2003 bp	No band	Neo targeted
PCR (P3.fwd + P3.rev)	1191 bp	2182 bp	230 bp	Neo deleted

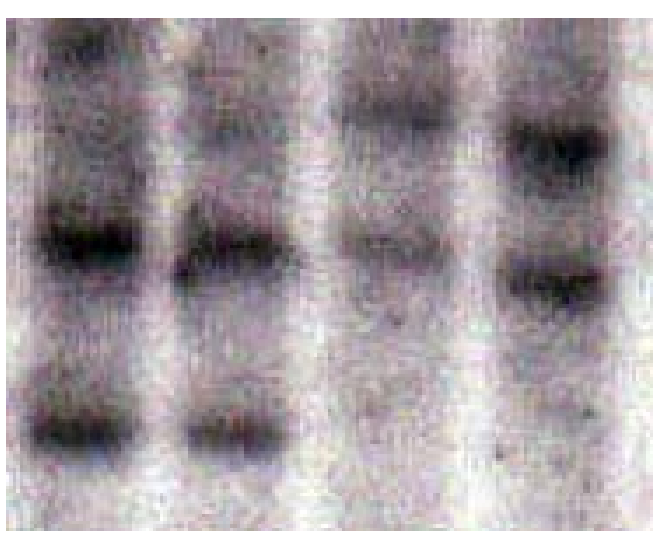

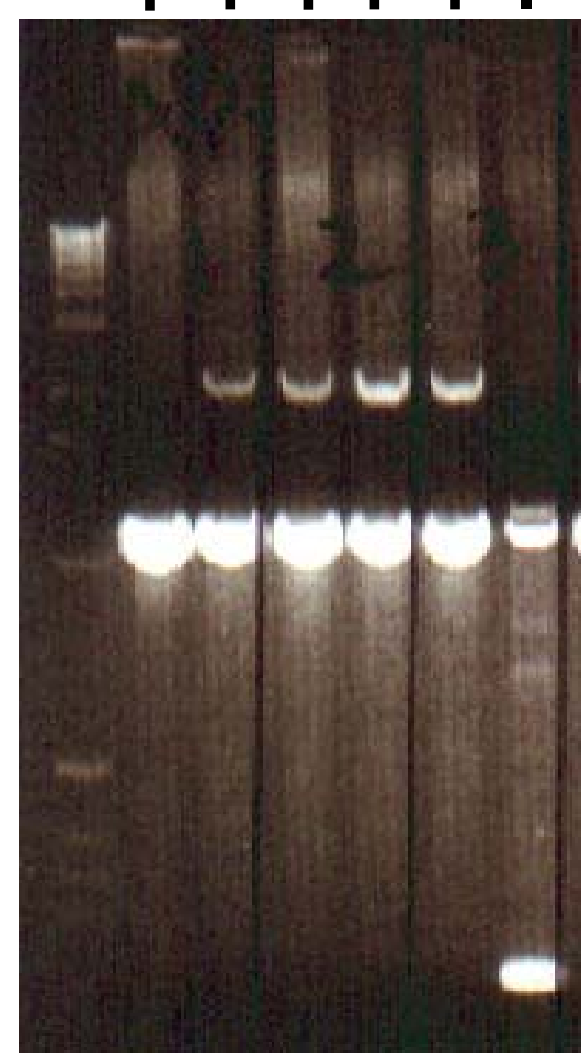

uc329 Primers

	Primers	Size (bp)
Long arm	5': gaatgcggccgcgagctgctcatgtatgactgagct	5047
	3': gtagctcgagcagcaggataaggcagaaattagg	
Short arm	5': ccatcggatccactgacagaaaggcaggctagg	1870
	3': ccatcgaattcctcccacaccgttctgtgatt	
Probe	5': ggctattgttataaaaagaggttagcac	256
	3': ctctgtgtggtcatttctattccc	
Neo target screening	5':gctatgctggacagacctcagtctt	Neo-2003<br>WT-none
	3': ttggctggacgtaaactcctctt	
Neo removal	5':acgtactttcccatgctttcc	Neo del-230<br>Neo-2182<br>WT-1191
	3': gatagctctcctgtgagtcagtagg	
Genotyping	5':acgtactttcccatgctttcc	Del-230<br>WT-320
	3': gatagctctcctgtgagtcagtagg	
	3': acagagcgtgattagcacag	

uc467 deletion

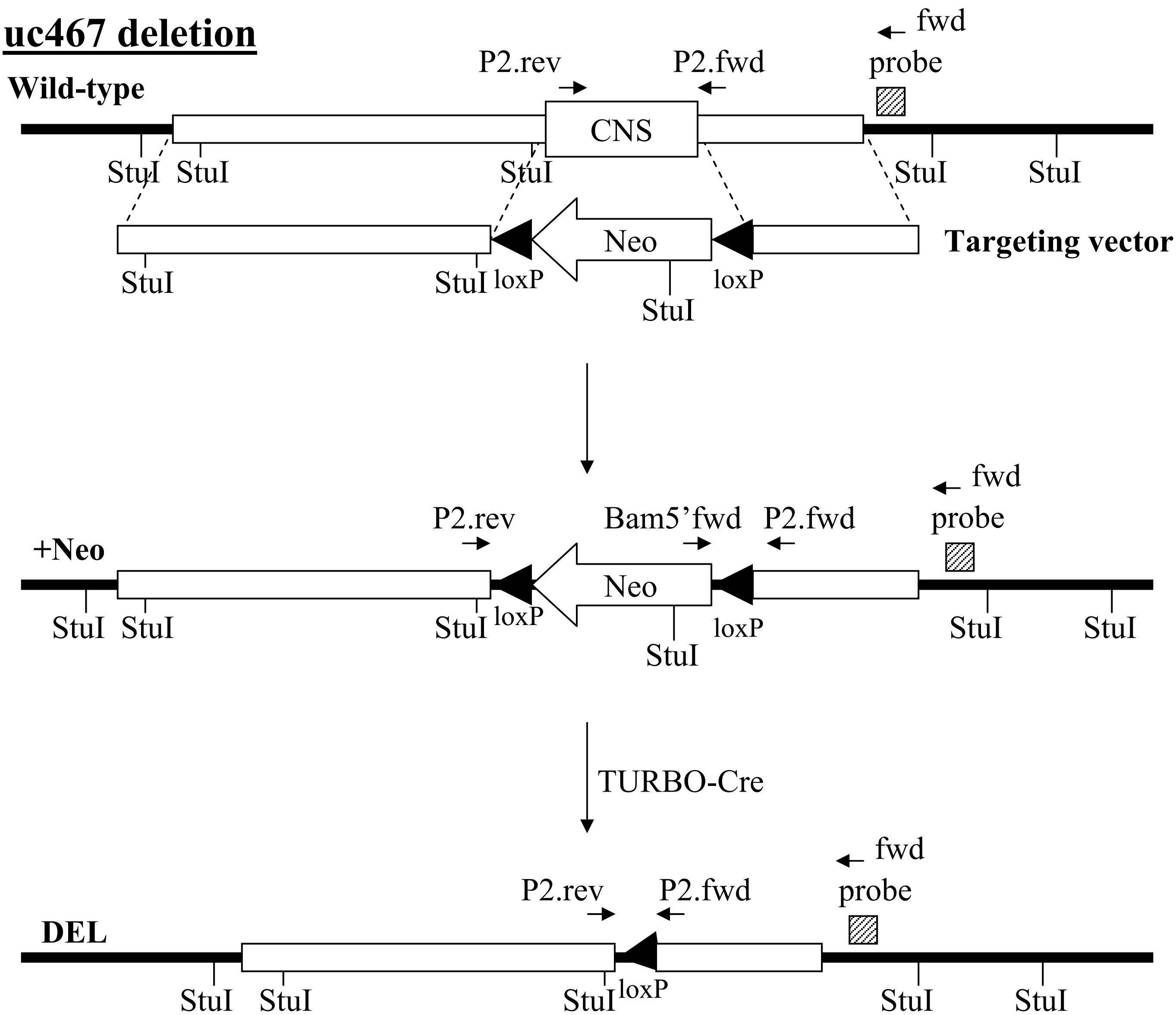

-Figure legend on page 2.

uc467 deleted sequence

acttgaggagggaggagacagggcgaggcgatgtaaagaatgccatgtctatggagaaaaaatagaaagtgagagtcgtgccccttaccatgtttcagt
ggagaaagagtcaccttgaaagagatcaaaaatttcctttcaactggatgaagtgattctatcagggttgggttttgggtgtttgtatttgtcatgtcggaagaa
aattgcccatgcagccccccttgctgaggttataaaaagtactgtcaaccatactggacagaattttttttttggaaggggggtgcggagaaggggaaaaca
cattgtgcaggatcattaagcactgttgtataaataaagttagaagatcaaacgaaagaggggactcgggttaaatgctggaagacaagcttaaataaagtt
ctaagaaaaaagcaaggcacgctgtaattccaagaacctattacttcagacaatttacttcaaaaaataaaatcagaatggtaaatgattacccaaaagcaa
tcatgtcatcatcactgagagatggaataaaataatttttttgtgaagtggctgaatgagagcaacttaaaagccacggagcctgtcagaagcacattatttctt
tttacaccccgtttcccaattcatctcagtgctgctgactaacctcggctcccagcaggtggagcatttaaaataaatagcatttcctacaaaacgcgctcca
actgccccatgacagacgcttccaattcccggccagccactggatcagcttcagcggtatctaacaaggtgtaaatacaataacaagcatgtaattaagtg
agcatgatgaagttaatggagataattcattccattttgggcttatgaatattctattagatgttatcaggcagctggaatagctgttaatttaatcatcattcaga
ccagcaaaatgttctttgtgcgagcataattgcagagaatgaataattgatttgacaattgtaccagtggatttcaaacagttctgtgcgctctcagagcagga
agggaggagaactggaatactcaaagcactgaagaggtggcagagaagccagcctgtaaattgaacattatcaggacacagataaaggacaatttttatt
ttatcaatgcaacacaattacattacagtgcgctagtaatcattctgcccacactttaaaaagggaaataaattactcttcatgggaaaggacaaaaagaaat
agcaaaaccatttagaatccattgatattggatatttaattttttgcattatattttattataggaaatatcaaactgtgtttgaaattgctggcacatttaactctgtta
cctgcaatcaggtacacagattgtattttacacgtatctctgtattacagcatgttctatgaattacaatgactttattgcaatttctttaaaaactcccctaatgttt
aaaaagagccatcaatgtggcatacaagagtactgtatgtacaccattaactatcaggactcggctctcgttacattcaacaactagcttttccccctaatggt
tctatactgtatgtcgatggaaaaagtagcaacaaaatattgatct

Southern or PCR	Wild-type	Neo targeted	Neo deleted	Screen for
**StuI digestion**	**4188 bp**	**2991 bp**	**2669 bp**	**Both**
**PCR(Bam5'.fwd/fwd)**	**No band**	**2149 bp**	**No band**	**Neo targeted**
**PCR (P2.fwd + P2.rev)**	**1746 bp**	**2179 bp**	**227 bp**	**Neo deleted**

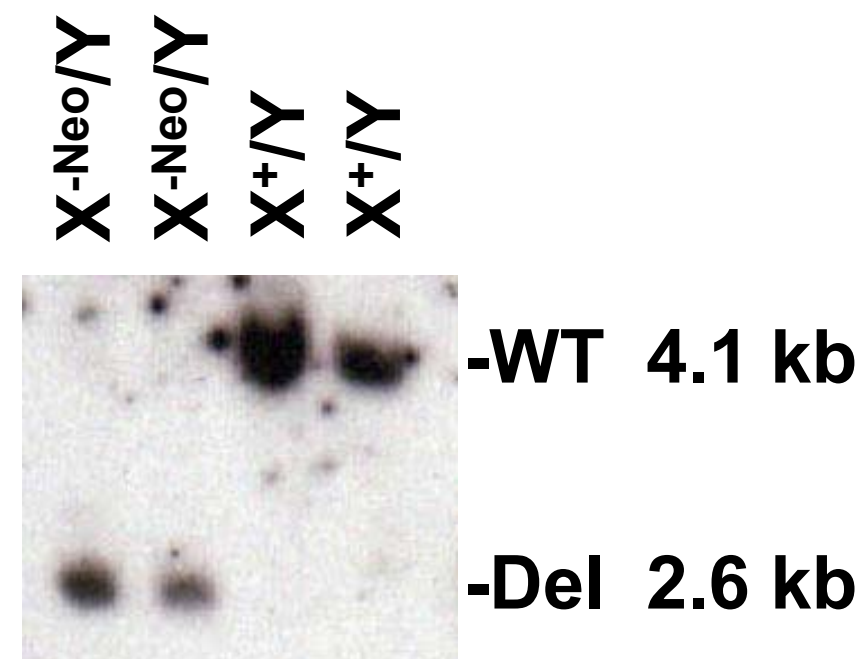

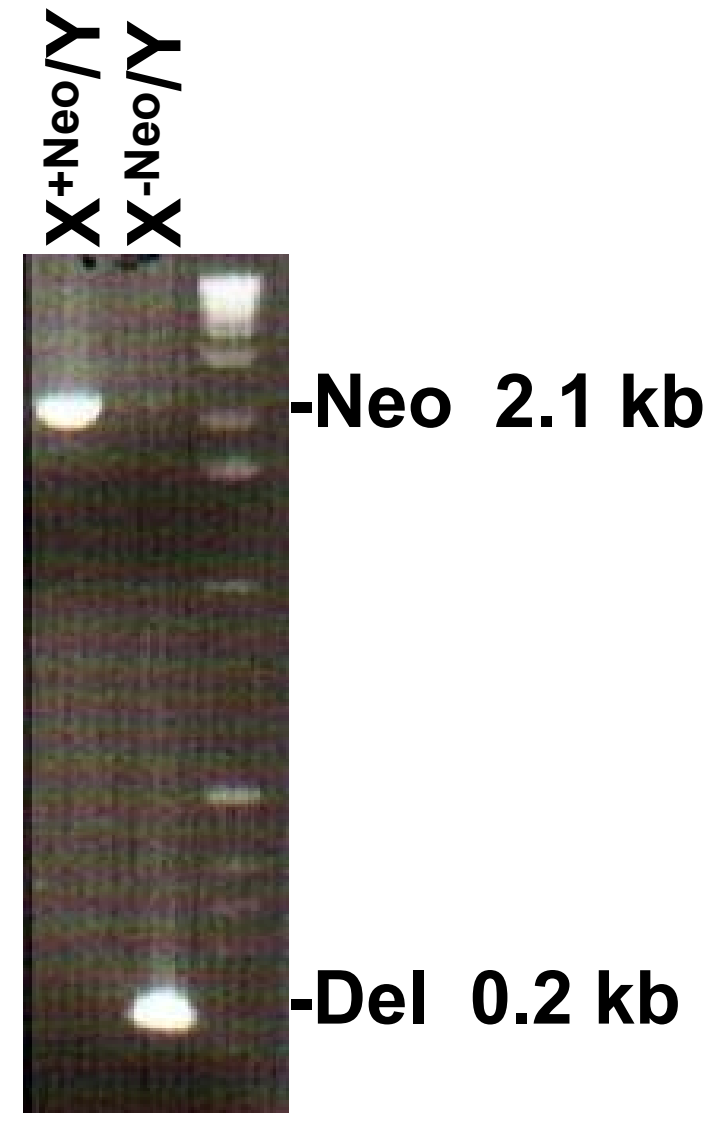

uc467 Primers

	Primers	Size (bp)
**Long arm**	**5': gaatgcggccgcgttcatgcgttagtgtgtttaagtgtggta**	**5261**
	**3': gtagctcgagtcaatgagccatctctgtcatgaaactcag**	
**Short arm**	**5': ccatcggatcccaccctctgctcttttccaaacttcc**	**2021**
	**3': ccatcgaattcactgttacctggtttctgtcaatattctacc**	
**Probe**	**5': agaggacatagctcctgatgtgtttac**	**326**
	**3': tatgatgtggcctgtgacagcttatc**	
**Neo target screening**	**5': gggcatccatgttaggctttacact**	**Neo-2149<br>WT-none**
	**3': ttggctggacgtaaactcctctt**	
**Neo removal**	**5': tacttgaagcaaatggaggtgcaatg**	**Neo del-227<br>Neo-2179<br>WT-1746**
	**3': ctaggcctttggtagagttgatccat**	
**Genotyping**	**5': tacttgaagcaaatggaggtgcaatg**	**Del-227<br>WT-348**
	**3': ctaggcctttggtagagttgatccat**	
	**3': ttctgtccagtatggttgacagtact**	

uc482 deletion

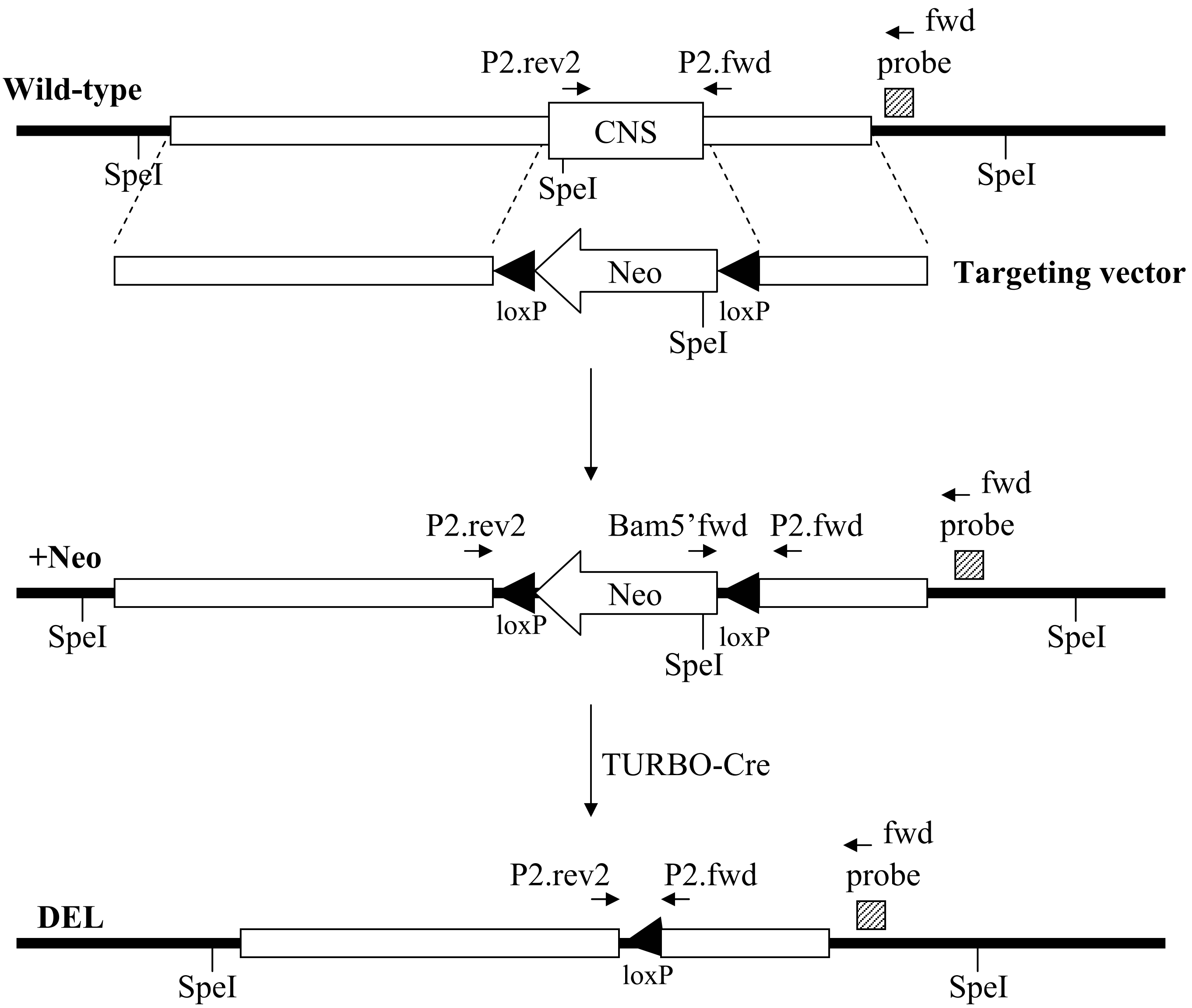

-Figure legend on page 2.

Southern or PCR	Wild-type	Neo targeted	Neo deleted	Screen for
SpeI digestion	5830 bp	3079 bp	7626 bp	Both
PCR(Bam5'.fwd/fwd)	No band	1936 bp	No band	Neo targeted
PCR (P2.fwd + P2.rev2)	3596 bp	2096 bp	144 bp	Neo deleted

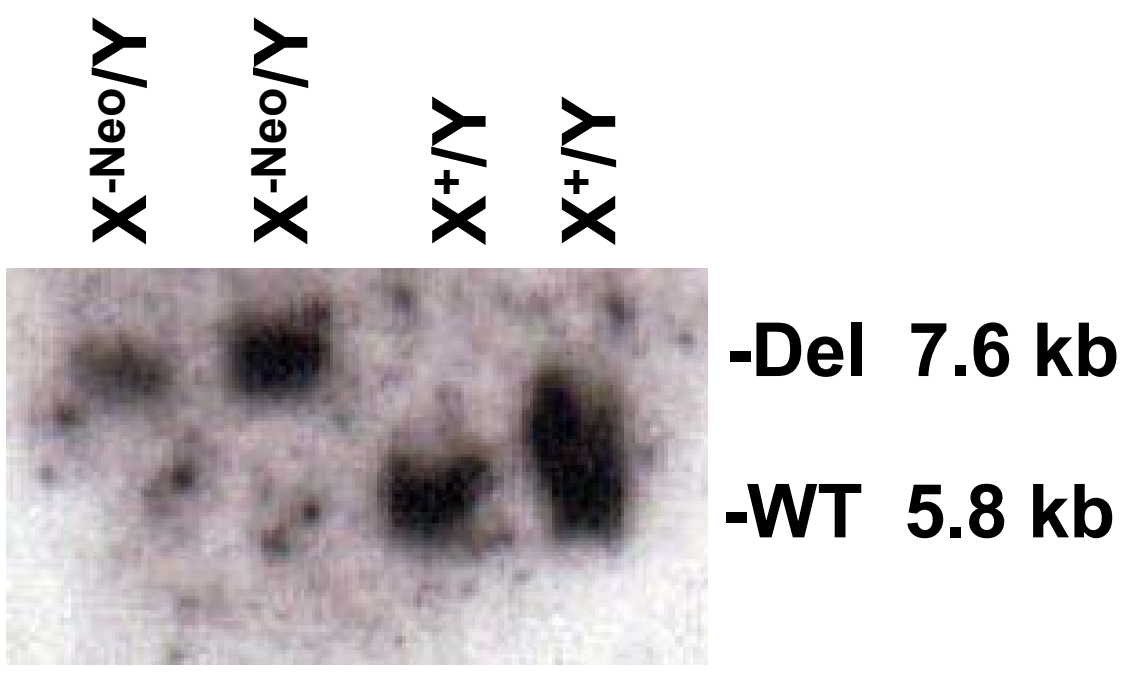

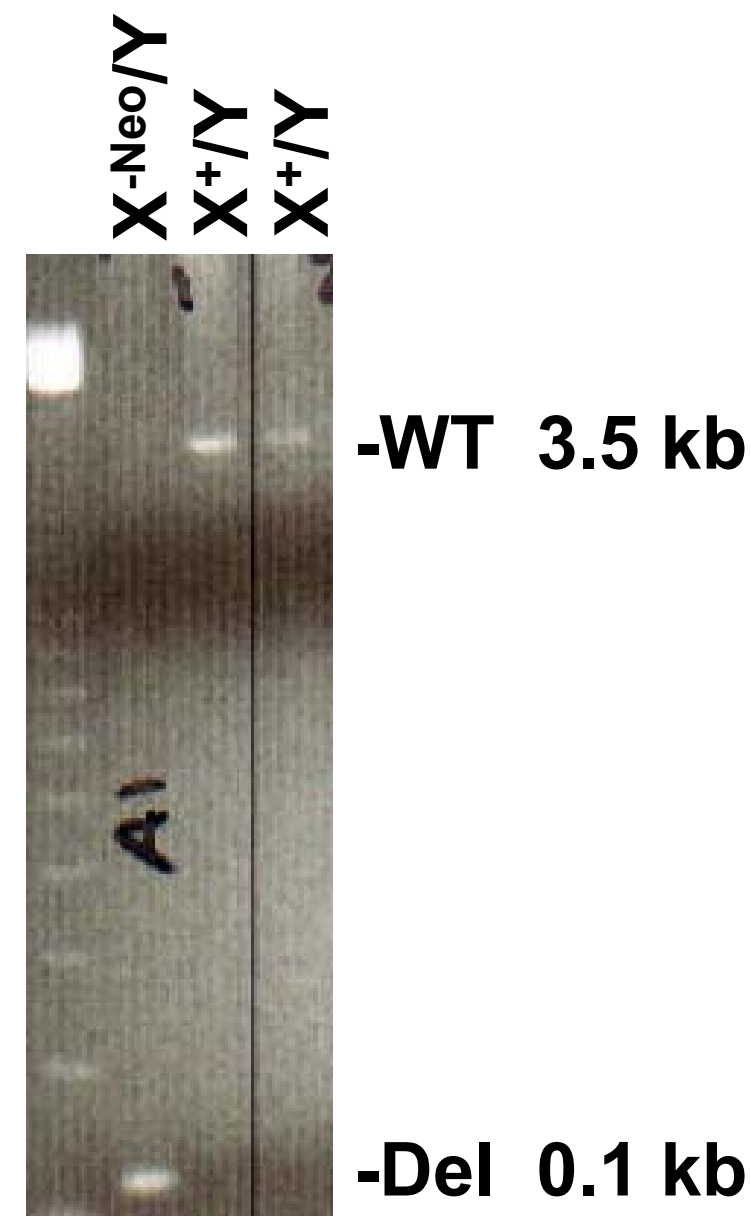

uc482 Primers

	primers	Size (bp)
Long arm	5': gaatgcggccgctgggaacaaatctgatgtgaacttagaggc	3119
	3': gtagctcgagccagctcatttggaagaagctgacaagtcca	
Short arm	5': ccatcggatcctctgtcttagtttgaggttctgtaaatagt	1730
	3': atcggggtacctccattagtttgaatgctaaccagaaag	
Probe	5': cagagaaatacatatattgctgtttctc	255
	3': gagacctttatttttatttacccctcta	
Neo target screening	5': ggaagtggggtatggcattagag	Neo-1936<br>WT-none
	3': ttggctggacgtaaactcctctt	
Neo removal	5':actatttacagaacctcaaactaagacaga	Neo del-144<br>Neo-2096<br>WT-3596
	3': tggacttgtcagcttcttccaaatgag	
Genotyping	5':actatttacagaacctcaaactaagacaga	Del-144<br>WT-305
	3': tggacttgtcagcttcttccaaatgag	
	3': ttattgctttcatgtgcctccacc	

uc482 deleted sequence

aaagaagtaacaaactttgtaaaaaatagaactattcttctctctctgcatatggtttaatattttccctgaagtaaaactttcagtctgtgaccttgagaaaccca
gaaagtaaatgcaaacagtacaggacggaaggtcacccatcctctctactactttccataaagggtatcaaatattcatccgcacaggtacatgtcttcgta
ataaggcagatgggaactaaagcaaaggcaactccaaatgggggtggaggcacatgaaagcaataattttgaatctttgacatttgttttgaaactacctta
ctaccctataccctttaccatctgtctgttatccctcccaacacccacctctgtcttcccacagggctccattgtctgcagttcctgtttctcaggggcggagct
gtgggaggcagccatgggtgccataataggcacctgcggctagcacaatgggctgtgggtgcttgtgggagagacagattgatgtattgtgcatggggg
aggagggctgtcacttgaatccatgtgaacccaggctttaggggcaagggagattgacagcttttgtaggttgcatttctttttcaaagcttggctttaataga
cagtaagtttggggccccaggctttcactcccataattctaacagggcaatcacgagcattcagcatttctttttaccccccttgttccgcattaaatgatttca
gtaaaggaatgtgcagcattaattagaatttgttagcagataccaaggggaaaagtttcttcacaacaactataggaggtgtcatttgtacaaagactttttgt
tttctttccccattacattatcactgcaatatgtaaggtagtgattcttttcatttaaattaagtggaaagctactaaatcttgttttattctccaaaaagaataaggtc
cttaacacttatcttttaacttctttcatttcctaaggttggtgtttacttttgctttcagtccaacccatttactggtctgtgaggttgtgtatccagttttgtgtaccatt
gagctcttacaattgcaggcagcccactgtactgatatagaaagattgtttccagggaaaaatggccacaaataatattggaaaacattgatataccagtcc
ctttttagtaatggtacatcaagaagtgccaaaaattagagtcatttaggttcatatcctagtgttttcaaaagccattttaaccttacccataacatcaaagttat
ggaaggatggggcagtggccaaagaatgtttttttttttcctatagaaaactcattccgaaactacgtaaaaaattttaagcatcatcctcacactcgcatagc
accattttcttacagttagtaaaaaggatctctctactttgtgtctatgttttattctttgacatctatctagtatgaaatctaatgtcagatagacatgtcctataagc
atgttcttcagtgtttggaaaactgatgaaacgttattaacatttttgatgtgattcttctagcatctctcctgtataaaatcatttcccaccaatgggaagagttga
aggctaattgaattagatttttttcatttaattcttttacttaaatgtgtcaggacagcagtgaaaaatattaagtctatcctgaattctcagagagaaaatattccca
gagttgtctaactctgtctataacaatttggtgcaatggctttaaaagtagtggggtgataataattagggtgtgtgacagaaatttcagtgttggattcaacatt
ttactgtgcagggcatgaaaattgccaagtgggggctagtcagctctaagtactaattaactaattaatcaggtgctgaaaccaagtgaatggaggatacca
gccagacaactcaagcccaagcagagggttgcactctttaaaagacactgcactctttcttcttggtttttaacccaatcaggaatgagagagaggcgggg
gaggggctaggtgggggtgggtgggttatggtacaagcttttcacttaggaaaaagtaagataaaaacatgtgcctctgataatgctgtaaggcgccctga
gtaggtctgtggctgactttccagtcaagaagtgtttttggtttggtgggagctggaggtatagagaattctgtcttcctggggtttatgtctataatacatacat
atatatcatatacatcatatacatctacatctacatgtatacatgtaggagaatgagaaacatgtctcttggggagctgatcttaggtcttttgcctcatccttcgt
ttatgtaaatgagaagcgtccaattacacatatggaaaaaaggtttaatgaatcgtgaaaagcctggtttgtagagaaagggcacagtctcttctccttatttc
cctcctcctcctcctcctcctcctcctcctcctcctcctctacctcctgttcatccacctcccttacttctccctcttgaccttcagatttcatttagaaacatgt
atttcttttcaggatgatgatgctcatcatttttttcacattctaattttaatacttaagaattatcaaacactttcctagaagattgaaatttatatttgtaaggttttctg
gatacttaaaatattctacactagaaactacaaaattctgattttcctcccctaaatttgaaaaaaaaaaagattacatgtctgttcctccccccctttgatgttattc
ttggctcaactttaatttcatgttttgtttgttctggaattgcttgcagtgaaaatgggttgcaacattagaaaggaactaatactgagtccaaagaaaagacagt
cccccataacttgcagcttagtttggtattgcattttaactagctggaattttagggtgttggaaattaggcaattcttattttactttcttttcaaaaagagtccgaa
ttttctgctgcagtagaaagttctttttttagttttcagttattttctttctccttttcttaatgttggtgtcctgagtagttggaagtggatcacacagaaccccttacca
tcccaagttttaaaatgatacaaacctttacagtaagactaaggagatcagtgctatgtctggccatccctctcagaatctgagaagtatgcaactagttccaa
agaagtattgtgagagaacagcagggcgcttcgcaatcatgaccttcaagtctttctcagagaccttcaagtcttcctcagagacaaggttcctcactgggc
atctggggaaactgaggcctaagaggacgagaaaggagcagttaccttttctccctcgacaaaatactgaccgaattatgccacgtgtttcttctaagtgta
gaagccaaagggtccatatcgcctgtctagttcactaaagggacgggtcttccactctacccctcagcctccctaatcagcaccccagatgtgaaaaacaa
cagaaagttgctcccctaatcctcagtgacaacacggggctgtaaaatttca

References

1. Paszty C, Mohandas N, Stevens ME, Loring JF, Liebhaber SA, et al. (1995) Lethal alpha-thalassaemia created by gene targeting in mice and its genetic rescue. Nat Genet 11: 33-39.
