# Supplementary material for: Deletion of Ultraconserved Elements Yields Viable Mice
Source: PLoS Biol. 2007 Sep 4;5(9):e234. doi: 10.1371/journal.pbio.0050234 (PMC1964772; doi:10.1371/journal.pbio.0050234)
Supplement: Supplementary file 2 [file pbio.0050234.st001.pdf]

Supplementary Table 1. Summary of pathological analysis in ultraconserved knockout mice

LINE	Sex	Age	Kidneys	Pancreas	Liver	Spleen	Lung	Heart	Thymus	GI Tract	Reproductive tract	Gonads	Urinary Bladder	Brain	Nasal cavity	Teeth	Eyes	Comment
**Wild-type**	M	6 weeks	NSL*	NSL	NSL	NSL	NSL	NSL	NSL	NSL	Preputial glands, prostate, seminal vesicles, and coagulating gland - NSL	Both testes - NSL	NSL	NSL	NSL	NSL		No significant changes were evident in this mouse
**Wild-type**	M	6 weeks	NSL	NSL	NSL	NSL	NSL	NSL	NSL	NSL	Preputial glands, prostate, seminal vesicles, and coagulating gland - NSL	Both testes - NSL	NSL	NSL	NSL	NSL		No significant changes were evident in this mouse
**Wild-type**	F	6 weeks	NSL	NSL	NSL	NSL	NSL	NSL	NSL	Glandular portion of stomach has a small focal pocket of ring form neutrophils on the serosal surface. The submucosa immediately adjacent to this focus contains similar inflammatory cells. The overlying mucosa is unremarkable.	Uterus, vagina, and mammary gland - NSL	Both ovaries -NSL	NSL	NSL	NSL	NSL		Focal gastritis - incidental finding.
**Wild-type**	F	6 weeks	NSL	NSL	NSL	NSL	NSL	NSL	NSL	NSL	Uterus, vagina, and mammary gland - NSL	Ovaries not examined.	NSL	NSL	NSL	NSL		No significant changes were evident in this mouse.
**uc248**	M	6 weeks	NSL	NSL	NSL	NSL	NSL	NSL	NSL	NSL	Preputial glands, prostate, seminal vesicles, and coagulating gland - NSL	Both testes - NSL	NSL	NSL	NSL	NSL		No significant changes were evident in this mouse
**uc248**	M	6 weeks	NSL	NSL	NSL	NSL	NSL	NSL	NSL	NSL	Preputial glands, prostate, seminal vesicles, and coagulating gland - NSL	Both testes - NSL	NSL	NSL	NSL	NSL		No significant changes were evident in this mouse
**uc248**	F	6 weeks	NSL	NSL	NSL	NSL	NSL	NSL	NSL	NSL	Uterus, vagina, and mammary gland - NSL	One ovary examined - NSL	NSL	NSL	NSL	NSL		No significant changes were evident in this mouse.
**uc248**	F	6 weeks	NSL	NSL	NSL	NSL	NSL	NSL	NSL	NSL	Uterus, vagina, and mammary gland - NSL	Both ovaries -NSL	NSL	NSL	NSL	NSL		No significant changes were evident in this mouse
**uc329**	M	6 weeks	Right kidney** - NSL	NSL	NSL	NSL	NSL	NSL	NSL	NSL	Preputial glands, prostate, seminal vesicles, and coagulating gland - NSL	Both testes - NSL	NSL	NSL	NSL	NSL		Agenesis of left kidney.**
**uc329**	M	6 weeks	NSL	NSL	NSL	NSL	NSL	NSL	NSL	NSL	Preputial glands, prostate, seminal vesicles, and coagulating gland - NSL	Both testes - NSL	NSL	NSL	NSL	NSL		No significant changes were evident in this mouse
**uc329**	F	6 weeks	NSL	NSL	NSL	NSL	NSL	NSL	NSL	NSL	Uterus, vagina, and mammary gland - NSL	One ovary examined - NSL	NSL	NSL	NSL	NSL		No significant changes were evident in this mouse
**uc329**	F	6 weeks	NSL	NSL	NSL	NSL	NSL	NSL	NSL	NSL	Uterus, vagina, and mammary gland - NSL	Not evident in sections examined from the cranial poles (Ovarian region) of the uterus.***	NSL	NSL	NSL	NSL		Ovaries were not evident. No other significant changes were evident in this mouse.***
**uc329**	F	6 weeks										Both ovaries had follicles in various stages of development - NSL						Ovaries were normal.
**uc329**	F	6 weeks										Both ovaries had follicles in various stages of development - NSL						Ovaries were normal.
**uc329**	M	7 weeks															Both eyes were unremarkable.	Eyes were normal.
**uc329**	M	7 weeks															Both eyes were unremarkable.	Eyes were normal.
**uc329**	F	7 weeks															Both eyes were unremarkable.	Eyes were normal.
**uc329**	F	7 weeks															Both eyes were unremarkable.	Eyes were normal.
**uc467**	M	6 weeks	NSL	NSL	NSL	NSL	NSL	NSL	NSL	NSL	Preputial glands, prostate, seminal vesicles, and coagulating gland - NSL	Both testes - NSL	NSL	NSL	NSL	NSL		No significant changes were evident in this mouse
**uc467**	M	6 weeks	NSL	NSL	NSL	NSL	NSL	NSL	NSL	NSL	Preputial glands, prostate, seminal vesicles, and coagulating gland - NSL	Both testes - NSL	NSL	NSL	NSL	NSL		No significant changes were evident in this mouse
**uc467**	F	6 weeks	NSL	NSL	NSL	NSL	NSL	NSL	NSL	NSL	Uterus, vagina, and mammary gland - NSL	One ovary examined - NSL	NSL	NSL	NSL	NSL		No significant changes were evident in this mouse
**uc467**	F	6 weeks	NSL	NSL	NSL	NSL	NSL	NSL	NSL	NSL	Uterus, vagina, and mammary gland - NSL	Not evident in sections examined from the cranial poles (Ovarian region) of the uterus.***	NSL	NSL	NSL	NSL		Ovaries were not evident. No other significant changes were evident in this mouse.***
**uc467**	F	6 weeks										Both ovaries had follicles in various stages of development - NSL						Ovaries were normal.
**uc467**	F	6 weeks										Both ovaries had follicles in various stages of development - NSL						Ovaries were normal.
**uc482**	M	6 weeks	NSL	NSL	NSL	NSL	NSL	NSL	NSL	NSL	Preputial glands, prostate, seminal vesicles, and coagulating gland - NSL	Both testes - NSL	NSL	NSL	NSL	NSL		No significant changes were evident in this mouse
**uc482**	M	6 weeks	NSL	NSL	NSL	NSL	NSL	NSL	NSL	NSL	Preputial glands, prostate, seminal vesicles, and coagulating gland - NSL	Both testes - NSL	NSL	NSL	NSL	NSL		No significant changes were evident in this mouse
**uc482**	F	6 weeks	NSL	NSL	NSL	NSL	NSL	NSL	NSL	NSL	Uterus, vagina, and mammary gland - NSL	One ovary examined - NSL	NSL	Not examined	NSL	NSL		No significant changes were evident in this mouse
**uc482**	F	6 weeks	NSL	NSL	NSL	NSL	NSL	NSL	NSL	NSL	Uterus, vagina, and mammary gland - NSL	Both ovaries - NSL	NSL	NSL	NSL	NSL		No significant changes were evident in this mouse

***NSL= No significant lesions or changes.**

****An additional 102 uc329 homozygous knockout mice were analyzed finding 2 mice, including this one, with unilateral renal agenesis.**

*****Ovaries were invertibly lost due to their small size during histological processing; we thus analyzed another 2 homozygous females to confirm presence of normal ovarie**
